# Management of Persistent Hyponatremia Induced by Long-acting Injectable Risperidone Therapy

**DOI:** 10.7759/cureus.2657

**Published:** 2018-05-20

**Authors:** Waliul Chowdhury, Muhammad Uzair Lodhi, Aaron R Kuzel, Peter Johnson, Umar Rahim, Mustafa Rahim

**Affiliations:** 1 Medical Student, Department of Medicine, Raleigh General Hospital, Beckley, Wv; 2 Department of Emergency Medicine, Lincoln Memorial University-Debusk College of Osteopathic Medicine; 3 Lincoln Memorial University, Raleigh General Hospital, Beckley, Wv; 4 Pre-Medical Student, Department of Sciences, Queens University of Charlotte, Nc; 5 Assistant Clinical Professor of Internal Medicine, West Virginia University School of Medicine

**Keywords:** family medicine, psychiatry, siadh, hyponatremia, tolvaptan, demeclocycline, management of persistent hyponatremia, preventive medicine

## Abstract

We present the case of a 55-year-old Caucasian male with a history of schizophrenia presenting with severe hyponatremia attributed to long-acting injectable risperidone treatment. Antipsychotic-induced hyponatremia is an uncommon but serious side effect that should be considered when assessing individuals on chronic psychiatric regimens. In this report, we will discuss our treatment plan for the patient when water deprivation and hypertonic saline failed to correct his serum sodium levels. The goal of this case report is to raise awareness of severe hyponatremia as a side effect of long-acting risperidone, and to encourage further studies to create guidelines for its management when current protocols fail to correct sodium levels.

## Introduction

Risperidone is a long-acting second-generation antipsychotic drug which is generally well tolerated with few side effects [1]. However, hyponatremia secondary to syndrome of inappropriate antidiuretic hormone (SIADH) is a well-known associated adverse effect of these medications [2]. If water deprivation and hypertonic saline fail to correct the patient’s hyponatremia, treating SIADH in-patients with tolvaptan compared to demeclocycline could be a more effective approach [3]. Cases of antipsychotic-induced hyponatremia have been reported in the literature. However, long-acting depot injections of risperidone causing hyponatremia have been seldom reported. Also, there are currently no clear guidelines as to how to manage patients with SIADH from long-acting risperidone treatment and who fail to recover with water deprivation and hypertonic saline. This was the reason for presenting this case report. The dilemma in our case was that we were not able to correct hyponatremia from long-acting risperidone therapy after the failure of water restriction and hypertonic saline. Discontinuing risperidone in this case also showed no acute benefit as its half-life is four to six days in the body [4]. We will also discuss the effectiveness of tolvaptan, as it was effective in our case when all other measures failed. 

## Case presentation

History and physical examination

A 55-year-old Caucasian male presented to the emergency department with the complaint of fainting spells with associated dizziness that persisted for several days. The patient stated that he had no associated alleviating or aggravating factors and had a significant tobacco use history. The patient reported a significant medical history of chronic obstructive pulmonary disease (COPD), hyperlipidemia, congestive heart failure, and gastro-esophageal reflux disease (GERD). In addition, the patient reveals that he is receiving treatment for psychiatric disorders that include schizophrenia and anxiety disorder. His medications included pantoprazole 40 mg once daily, risperidone 50 mg of intramuscular injections every two weeks, atorvastatin 10 mg oral once daily, buspirone 10 mg oral twice daily, clopidogrel 75 mg oral once daily, metoprolol 25 mg oral once daily, and nifedipine 60 mg oral once daily. The patient denied any recent changes in medication or travel history. He denied any fever or chills, orthopnea, or paroxysmal nocturnal dyspnea (PND). In addition, he denied any recent weight changes, nausea, vomiting, diarrhea, melena, odynophagia or dysphagia, heartburn, or intravenous drug abuse. No other symptoms of arthritis, mouth sores or mouth ulcers, photosensitive rash, or redness or swellings in the small joints of the hands were reported.

Upon physical examination, the patient seemed to be in no acute distress. He did appear slightly confused upon questioning, but was oriented in time, place, and person with no signs of focal neurological deficits. The functioning of all cranial nerves was intact. The patient appeared to be euvolemic at the time of examination and vital signs were within normal limits. Pulmonary examination revealed diffuse expiratory wheezes in both the anterior and posterior lung fields. The rest of the physical examination was unremarkable. 

Hospital course

**Figure 1:**
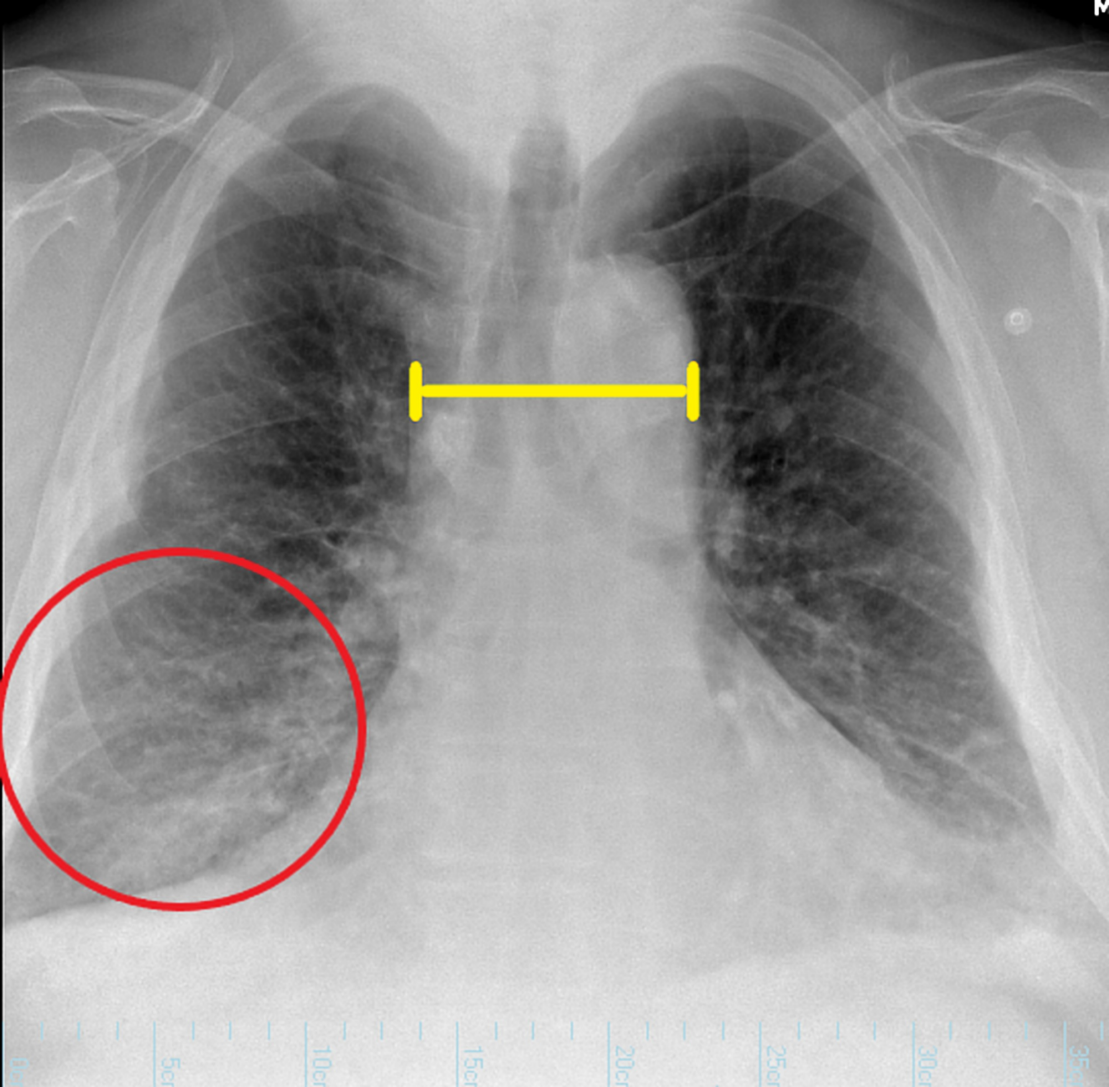
Chest X-ray, showing a right-sided lobar consolidation (red circle) and a widened mediastinum (yellow bar).

**Figure 2:**
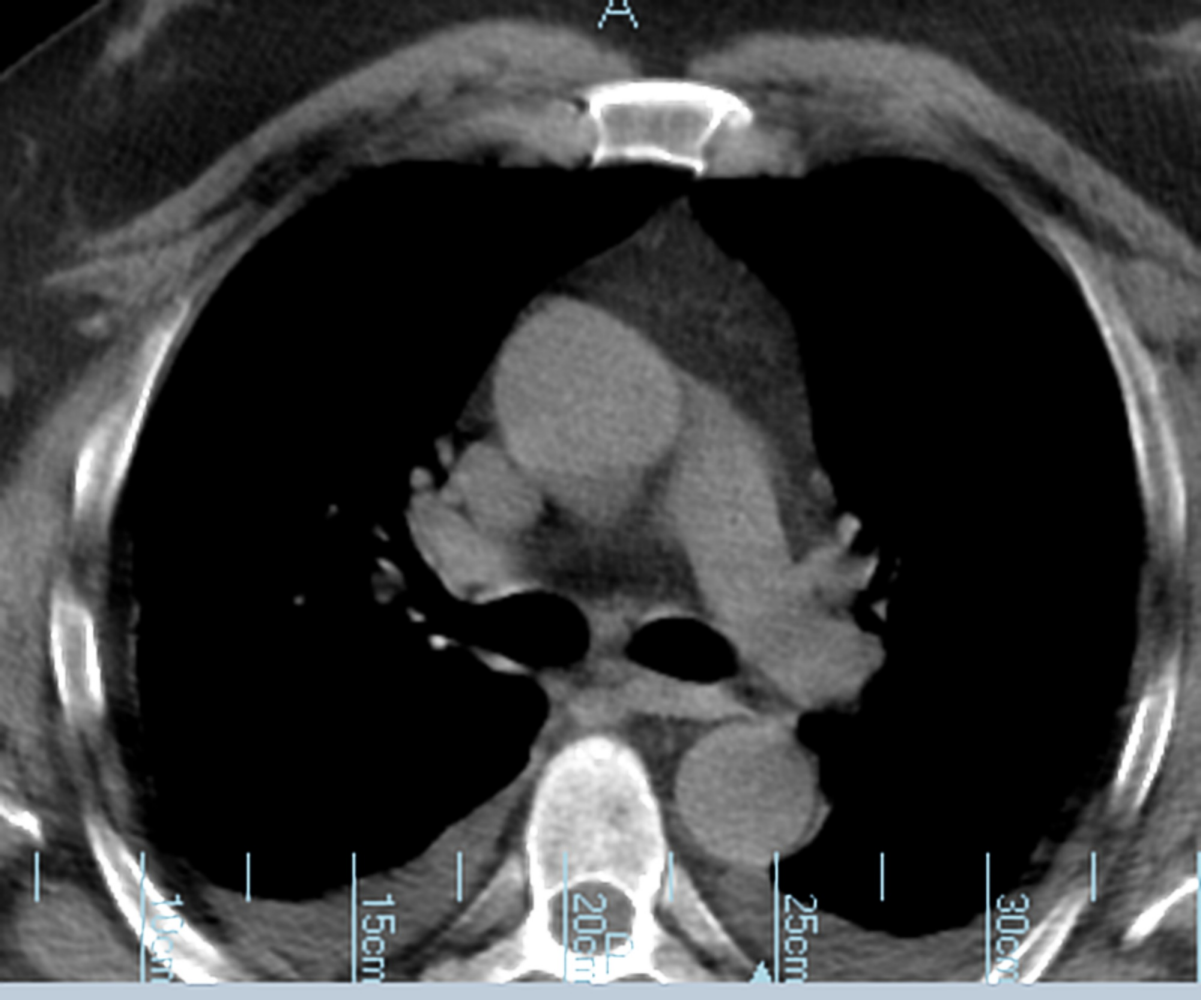
Noncontrast chest computed tomography scan, showing no abnormalities in the chest.

Laboratory workup of the patient revealed a critically low serum sodium level of 114 mmol/L. His serum osmolarity was 212 mOsm/kg and urine osmolality was 320 mOsm/kg. His urine sodium level was 63 mmol/L and serum uric acid level was 2.3 mg/dL. Given the patient's low serum osmolarity, high urine osmolarity, and urine sodium level greater than 40 mmol/L, these findings directed the medical team towards the diagnosis of SIADH. A chest X-ray was taken to look for any abnormalities, given his bilateral wheezing on physical examination. The findings showed a right-sided lobar consolidation and a widened mediastinum, as shown in Figure 1. A noncontrast computed tomography (CT) scan of the chest was clear of any abnormal masses, as shown in Figure 2. He was treated with ceftriaxone and azithromycin for pneumonia and his breathing difficulty reduced, but his hyponatremia still persisted. There was a high suspicion that his long-acting risperidone had contributed to this hyponatremia as an adverse effect, as he had been on chronic injections of risperidone for years. The medical team decided to withdraw the patient’s risperidone. However, this did not change his serum sodium levels as it was still severely low. This was expected because long-acting risperidone has a half-life of about four to six days in the body [4]. Water withdrawal and hypertonic saline were also initiated, but his serum sodium levels persisted at levels less than 120 mmol/L. Some 30 mg of tolvaptan was then administrated. Following the administration of 30 mg of tolvaptan, the patient’s serum sodium levels improved over the course of two days and subsequently remained above 130 mmol/L. His serum sodium level was 133 mmol/L during his two-week and one-month outpatient follow-up. This is still below normal, but it is definitely an improvement compared to his initial sodium levels, and his signs of mental confusion were not present on follow-up. 

## Discussion

Tolvaptan has been shown to have dose-dependent effects on water excretion in healthy volunteers up to a dose of 60 mg, after which a plateau effect develops [5]. Kim et al. conducted single and multiple dose studies to evaluate the pharmacokinetics, pharmacodynamics, and safety of tolvaptan in healthy Japanese volunteers. These studies were all randomized, placebo-controlled, and single or double-blinded. In the single-dose study, volunteers were given a single oral dose of either tolvaptan or placebo at 15–120 mg. In the multiple dose studies, the volunteers were given tolvaptan or placebo at doses of 30, 60, 90, or 120 mg once daily for seven days. After a single administration of tolvaptan at a dose of 15–120 mg, the maximum concentration of plasma (*C*_max_) and area under the curve (AUC) for plasma concentration versus time increased proportionately with higher doses. The increase in 24-hour cumulative urine volume was also dose-dependent. The maximum urine excretion rate reached a plateau at doses above 60 mg, but increased dose-dependently when doses were below 60 mg. A decrease in urine osmolality and an increase in the clearance of free water were seen in the subjects who received tolvaptan. Serum sodium concentrations were higher in subjects receiving tolvaptan compared to placebo, even one day after administration of tolvaptan. The most common side effect associated with tolvaptan was mild thirst [5]. This indicated that in comparison to placebo, tolvaptan effectively induces urinary excretion of water and increases serum sodium levels dose-dependently, with the effect reaching a plateau at doses greater than 60 mg.

Demeclocycline is of the tetracycline class of antibiotics and acts to block antidiuretic hormone (ADH) vasopressin-2 receptors in the distal collecting tubules of the kidney. In comparison, tolvaptan blocks ADH receptors in the renal tubules of the kidney [6]. The reason tolvaptan was selected in this case over demeclocycline was because tolvaptan has been shown to lower in-patient hospital costs and number of admissions compared to demeclocycline [2].

A retrospective cohort study by Grant et al. examined 3,060 patients who were hospitalized for SIADH. Demeclocycline was given to 927 of these patients and 120 patients were treated with tolvaptan. The rest of the patients were managed with fluid restriction, hypertonic saline, or no treatment. Patients who were treated with tolvaptan had shorter hospital stays and lower in-patient care expenses compared to patients treated with demeclocycline. However, demeclocycline was associated with lower outpatient appointments compared to patients on tolvaptan. This shows that the more cost-effective treatment option for in-patients with SIADH is tolvaptan followed by switching to demeclocycline upon discharge [2].

Before considering tolvaptan for treatment, patients should be checked for underlying liver disease. Torres et al. conducted a three-year double-blinded placebo-controlled trial on tolvaptan therapy in 1,400 patients with autosomal dominant polycystic kidney disease. They noticed that three patients treated with tolvaptan developed significantly increased levels of alanine aminotransferase (ALT) and serum total bilirubin. All three patients’ abnormal liver enzymes improved when tolvaptan was discontinued [7]. Although only three out of 1,400 patients in this study developed liver complications, it remains an adverse effect that physicians should be aware of prior to administrating tolvaptan.

The underlying association between second-generation antipsychotics like risperidone and hyponatremia could be multifactorial. In addition to risperidone causing SIADH, some data also suggest that risperidone could increase dopamine levels and reset the osmo-receptors in the brain causing polydipsia and dilutional hyponatremia [8]. This could be the underlying pathophysiology behind antipsychotics like risperidone causing SIADH as a side effect. Further research to better understand the underlying pathophysiology behind antipsychotic-induced hyponatremia is needed, in order to develop more tailored antidotes in the future.

## Conclusions

Long-acting antipsychotics are commonly used as a first-line treatment for chronic schizophrenic patients. However, hyponatremia is a serious adverse effect that physicians should be prepared for. Our case was unique because the patient’s hyponatremia was persistent despite discontinuing the long-acting antipsychotic and restricting fluids. When all attempts failed to increase the patients’ serum sodium levels in this case, a one-time 30 mg oral dose of tolvaptan was shown to be effective. It must be noted that tolvaptan should be avoided in patients with a history of underlying liver disease. Further studies are needed to develop specific guidelines on how to manage severe long-acting antipsychotic-induced hyponatremia that fails to respond to current management protocols like water deprivation. 
